# Microglial intracellular Ca^2+^ signaling as a target of antipsychotic actions for the treatment of schizophrenia

**DOI:** 10.3389/fncel.2014.00370

**Published:** 2014-11-05

**Authors:** Yoshito Mizoguchi, Takahiro A. Kato, Hideki Horikawa, Akira Monji

**Affiliations:** ^1^Department of Psychiatry, Faculty of Medicine, Saga UniversitySaga, Japan; ^2^Department of Neuropsychiatry, Graduate School of Medical Sciences, Kyushu UniversityFukuoka, Japan; ^3^Innovation Center for Medical Redox Navigation, Kyushu UniversityFukuoka, Japan

**Keywords:** microglia, calcium, endoplasmic reticulum, BDNF, proBDNF, antipsychotic, schizophrenia

## Abstract

Microglia are resident innate immune cells which release many factors including proinflammatory cytokines, nitric oxide (NO) and neurotrophic factors when they are activated in response to immunological stimuli. Recent reports show that pathophysiology of schizophrenia is related to the inflammatory responses mediated by microglia. Intracellular Ca^2+^ signaling, which is mainly controlled by the endoplasmic reticulum (ER), is important for microglial functions such as release of NO and cytokines, migration, ramification and deramification. In addition, alteration of intracellular Ca^2+^ signaling underlies the pathophysiology of schizophrenia, while it remains unclear how typical or atypical antipsychotics affect intracellular Ca^2+^ mobilization in microglial cells. This mini-review article summarizes recent findings on cellular mechanisms underlying the characteristic differences in the actions of antipsychotics on microglial intracellular Ca^2+^ signaling and reinforces the importance of the ER of microglial cells as a target of antipsychotics for the treatment of schizophrenia.

## Introduction

Microglia are immune cells which are derived from progenitors that have migrated from the periphery and are from mesodermal/mesenchymal origin (Kettenmann et al., 2011). After invading the brain parenchyma, microglia transform into the “resting” ramified phenotype and are distributed in the whole brain. However, microglia revert to an ameboid appearance when they are activated in the disturbances including infection, trauma, ischemia, neurodegenerative diseases or any loss of brain homeostasis (Aguzzi et al., 2013; Cunningham, 2013). Recent *in vivo* imaging has shown that microglial cells actively scan their environment with motile protrusions even in their resting state and are ready to transform to “activated” state in responses to injury, ischemia or autoimmune challenges in the brain (Wake et al., 2013). Microglia can release many factors including proinflammatory cytokines (such as TNFα, IL-6), nitric oxide (NO) and neurotrophic factors (such as BDNF) when they are activated in response to immunological stimuli (Kettenmann et al., 2011; Smith and Dragunow, 2014). In addition, microglia are shown to be involved in the development of neural circuits or synaptic plasticity thereby maintaining the brain homeostasis (Schwartz et al., 2013).

There is increasing evidence suggesting that pathophysiology of schizophrenia is related to the inflammatory responses mediated by microglia (Müller and Schwarz, 2007; Kato et al., 2011; Monji et al., 2013; Myint and Kim, 2014). A recent meta-analysis of associations between schizophrenia and dysfunction of immune systems including aberrant circulating cytokine levels showed that IL-1β, IL-6 and transforming growth factor-β (TGF-β) appeared to be state markers, as they were elevated in acutely relapsed inpatients or in first-episode psychosis and then normalized with antipsychotic medications. In contrast, IL-12, interferon-γ (IFNγ) and tumor necrosis factor α (TNFα) appeared to be trait markers, as they remained elevated in acute exacerbations of psychotic symptoms and even after the antipsychotic treatment (Miller et al., 2011). Microglial activation can be estimated by positron emission tomography (PET) using radiopharmaceuticals. For example, a quantitative (R)-[(11)C]PK11195 PET scan showed that activated microglia were present in the gray matter of patients suffered from schizophrenia within the first 5 years of disease onset (van Berckel et al., 2008). Another PET study using [11C]DAA1106 showed a positive correlation between cortical [11C]DAA1106 binding and positive symptom scores obtained from patients with schizophrenia (Takano et al., 2010). In addition, we and others have reported that pretreatment with antipsychotics significantly inhibits the release of proinflammatory cytokines and/or NO from activated microglial cells (Hou et al., 2006; Kato et al., 2013). Interestingly, pretreatment with haloperidol or risperidone significantly suppressed the release of proinflammatory cytokines and NO from activated microglial cells, although the inhibitory effects of risperidone were much stronger than those of haloperidol (Kato et al., 2007). In addition, we have previously shown that pretreatment with aripiprazole suppressed the elevation of intracellular Ca^2+^ concentration ([Ca^2+^]i) induced by IFNγ in microglial cells, suggesting the importance of microglial intracellular Ca^2+^ signaling as a target of antipsychotics for the treatment of schizophrenia (Kato et al., 2008; Mizoguchi et al., 2011), because elevation of intracellular Ca^2+^ is important in activation of microglial cell functions, including proliferation, release of NO and cytokines, migration, ramification and deramification (Färber and Kettenmann, 2006). Here, we briefly review our current understanding of the cellular mechanisms underlying the characteristic differences in the actions of antipsychotics on neuronal or microglial intracellular Ca^2+^ signaling and reinforces the importance of the endoplasmic reticulum (ER) of microglial cells as a target of antipsychotics for the treatment of schizophrenia.

## Schizophrenia and intracellular Ca^2+^ signaling

The electrical activity of neurons (i.e., excitable cells) depends on a number of different types of voltage- or ligand-gated ion channels that are permeable to inorganic ions such as sodium, potassium, chloride and calcium. While the former three ions predominantly support the electrogenic role, Ca^2+^ are different in that they can not only alter the membrane potential but also serve as important intracellular signaling entities by themselves. In the CNS, intracellular Ca^2+^ signaling regulates many different neuronal functions, such as cell proliferation, gene transcription and exocytosis at synapses (Berridge et al., 2003). In neurons, because the prolonged elevation of [Ca^2+^]i is cytotoxic, [Ca^2+^]i is tightly regulated by intrinsic gating processes mediated by voltage-gated calcium channels and NMDA receptors (NMDARs; Simms and Zamponi, 2014). In addition, dysregulation of neuronal Ca^2+^ signaling have been linked to various neuropsychiatric disorders including schizophrenia (Lidow, 2003). A possible involvement of intracellular Ca^2+^ signaling in schizophrenia was originally presented by Jimerson et al. (1979), based on their finding that remission from acute psychotic symptoms of schizophrenia was accompanied by elevation of the Ca^2+^ concentration in the cerebrospinal fluid. Thereafter, the interaction of neuronal dopaminergic transmission and intracellular Ca^2+^ signaling was documented. Dopamine D2 receptors were shown to be regulated by intracellular Ca^2+^ through the activation of CaMKII or neuronal Ca^2+^ sensor 1 (NCS-1). Both CaMKII and NCS-1 have also been reported to be involved in the pathophysiology of schizophrenia (Bai et al., 2004; Luo et al., 2014). Another topic of hypothesis underlying the pathophysiology of schizophrenia is the involvement of intracellular Ca^2+^signaling within the fast spiking GABAergic inhibitory neurons in the hypofunction of NMDARs which leads to the dysfunction of GABAergic inhibitory circuits (Lewis et al., 2005; Berridge, 2013). The sustained and synchronous firing of dorsolateral prefrontal cortical neurons in the gamma frequency range of approximately 40 Hz (gamma rhythms) depends on excitatory pyramidal neurons which release glutamate to activate the inhibitory GABAergic interneurons. The hypofunction of NMDARs results in the reduction of intracellular Ca^2+^ signaling, suppression of the induction of transcription factor CREB and reduction in the expression of the glutamic acid decarboxylase 67 (GAD67), which leads to the change of gamma rhythms and the impairment of cognitive functions observed in patients suffered from schizophrenia. In addition, dysregulation of the redox signaling pathway might provide an explanation for the developmental origins of schizophrenia because there appears to be a link between maternal viral infections during gestation and the incidence of schizophrenia. During viral infections, the increase of the IL-6 release and the resultant activation of redox signaling pathway promote the hypofunction of NMDARs in the GABAergic interneurons (Berridge, 2013).

Recently, there are many reports that have shown that possible involvement of single-nucleotide polymorphisms (SNPs) within two L-type voltage-gated calcium channel subunits, CACNA1C and CACNB2, and neuropsychiatric disorders including schizophrenia, suggesting that dysfunction of L-type voltage-gated calcium channels occurs in patients with schizophrenia (Ripke et al., 2013; Smoller et al., 2013). However, the activation of voltage-gated calcium channels are well known to be suppressed by the treatment of various antipsychotics (Santi et al., 2002; Choi and Rhim, 2010). For example, in cultured HEK cells, haloperidol acutely blocks T-type voltage-gated calcium channels in a dose-dependent manner (Santi et al., 2002), while it remains unclear whether antipsychotics also affect voltage-gated calcium channels in neurons. Solís-Chagoyán et al. (2013) recently reported that Ca^2+^ currents mediated by L-type voltage-gated calcium channels recorded in olfactory neuroepithelial cells obtained from patients with schizophrenia were 50% smaller than those from healthy subjects. Because these patients with schizophrenia were taking antipsychotics, the finding does not simply support the genetic studies suggesting that dysfunction of L-type voltage-gated calcium channels occurs in patients with schizophrenia.

## Antipsychotics and the ER-mediated microglial intracellular Ca^2+^ mobilization

Elevation of intracellular Ca^2+^ is also important for the activation of microglia, including proliferation, migration, ramification, deramification and release of NO, proinflammatory cytokines and BDNF (Kettenmann et al., 2011). However, in microglial cells, an application of high [K^+^]out or glutamate does not elevate [Ca^2+^]i. This observation is supported by the fact that both voltage-gated Ca^2+^ channels and NMDARs are not expressed in microglia (Kettenmann et al., 2011). For electrically non-excitable cells including microglia, the primary source of intracellular Ca^2+^ is the release from intracellular Ca^2+^ stores and the entry through the ligand-gated and/or store operated Ca^2+^ channels (Möller, 2002). Microglia contain at least two types of intracellular Ca^2+^ stores: the ER and mitochondria. The main route for the generation of intracellular Ca^2+^ signaling is associated with inositol 1,4,5-trisphosphate (InsP3) receptors on the ER membrane. Stimulation of G protein-coupled metabotropic receptors results in the activation of the phospholipase C (PLC), production of two second messengers including the diacylglycerol (DAG) and the InsP3 and the release of Ca^2+^ from the ER. Importantly, the depletion of ER activates the store-operated Ca^2+^ entry (SOCE), known as a capacitative Ca^2+^ influx, mediated by plasmalemmal channels such as calcium release-activated Ca^2+^ (CRAC) channels and/or transient receptor potential (TRP) channels (Parekh and Putney, 2005). In addition, STIM1, one of ER membrane proteins, senses the filling state of ER Ca^2+^ and delivers the ER to the plasma membrane where it directly activates Orai1/CRAC channels, thereby facilitating the re-uptake of Ca^2+^ to ER through the sarco(endo)plasmic reticulum Ca^2+^-ATPases (SERCA). The concentration of Ca^2+^ in the ER is precisely controlled by SERCA. The influx of Ca^2+^ through the TRP channels plays an important role in many inflammatory processes including the activation of microglia (Nilius et al., 2007; Mizoguchi et al., 2014). Because there is increasing evidence suggesting that pathophysiology of schizophrenia is related to the inflammatory responses mediated by microglia (Müller and Schwarz, 2007; Monji et al., 2013), it could be important to examine the effects of antipsychotics on the ER function of microglial cells for the treatment of schizophrenia.

**Figure 1:**
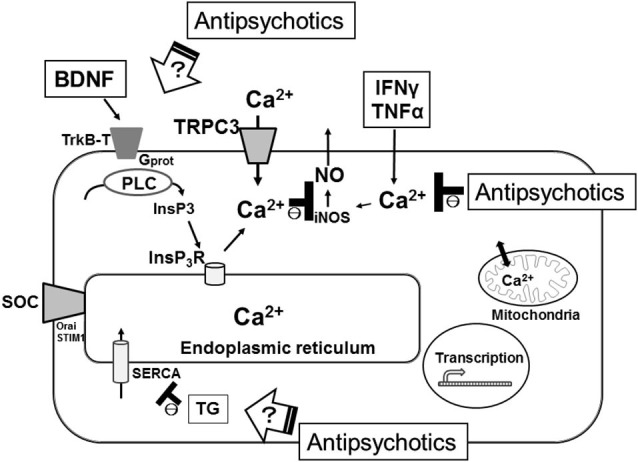
**Schematic illustration representing the microglial intracellular Ca^2+^ signaling, especially the ER function, as major targets of antipsychotics for the treatment of schizophrenia**. SERCA, sarco(endo)plasmic reticulum Ca^2+^-ATPases; TG, thapsigargin; SOC, store-operated calcium channel.

In some electrically non-excitable cells such as macrophages, adipocytes, β-cells and oligodendrocytes, perturbation of the calcium homeostasis in the ER results in the accumulation of unfolded proteins, the induction of the ER stress response, the promotion of the inflammatory processes and the initiation of apoptosis (Zhang and Kaufman, 2008). Experimentally, the ER stress response is frequently induced by selectively inhibiting SERCA using agents such as thapsigargin (TG) which passively deplete the ER (Thastrup et al., 1990). It remains unclear how typical or atypical antipsychotics affect the ER-mediated intracellular Ca^2+^ mobilization in microglia. Thus, we examined how pretreatment with typical (haloperidol) or atypical (risperidone) antipsychotics affects TG-induced intracellular Ca^2+^ mobilization, which represents a cellular stress response. In rodent microglial cells, we observed that opposite effects of haloperidol and risperidone on the TG-induced intracellular Ca^2+^ mobilization (Mizoguchi et al., unpublished observations). There are two other reports showing opposite effects of haloperidol and risperidone on intracellular Ca^2+^ mobilization. In cultured astrocytes derived from rat cortex and striatum, intracellular Ca^2+^ imaging showed that pretreatment with risperidone but not haloperidol suppressed the dopamine-induced increase in [Ca^2+^]i (Reuss and Unsicker, 2001). In another study obtained from rat PC12 cells, pretreatment with haloperidol potentiated the rotenone-induced neurotoxicity, while risperidone suppressed it. Likewise, pretreatment with haloperidol potentiated the rotenone-induced increase in [Ca^2+^]i, while risperidone completely suppressed it, suggesting that opposite effects of haloperidol and risperidone on rotenone-induced neurotoxicity could be mediated by their differential effects on intracellular Ca^2+^ mobilization (Tan et al., 2007). In addition, Kurosawa et al. (2007) reported that pretreatment with risperidone but not with haloperidol suppressed the death of rat cultured cortical neurons induced by treatment with TG for 72 h. Disruption of intracellular Ca^2+^ signaling triggers the activation of cell death programs (Orrenius et al., 2003). Treatment of primary cultured microglial cells by TG or ionomycin induced cellular apoptosis and this pathway was suppressed by the pretreatment with BAPTA-AM (Nagano et al., 2006). Thus, these suggest that typical and atypical antipsychotics have different effects on the ER-mediated intracellular Ca^2+^ mobilization, which might lead to the differences in the actions of typical and atypical antipsychotics on the induction of the ER stress response, promotion of the inflammatory responses and/or initiation of apoptosis in microglia (Figure 1).

Brain-derived neurotrophic factor is also well known for its involvement in the pathophysiology of neuropsychiatric disorders including schizophrenia (Autry and Monteggia, 2012). A recent meta-analysis of studies showed that blood levels of BDNF are reduced in both medicated and drug-naïve patients with schizophrenia (Green et al., 2011). In addition, expression of BDNF in rodent microglia is important for the spine elimination/formation and motor-learning processes (Parkhurst et al., 2013). We have recently reported that BDNF induces sustained [Ca^2+^]i elevation, which was mediated by an initial PLC/InsP3-driven Ca^2+^ release from the ER that followed by a long-lasting activation of the SOCE via the up-regulation of cell-surface TRPC3 channels in rodent microglial cells (Mizoguchi et al., 2009, 2014). In addition, incubation with BDNF decreased release of NO from the activated microglia, suggesting that BDNF might have an anti-inflammatory effect through the inhibition of microglial activation and could be useful for the treatment of neuropsychiatric disorders including schizophrenia. It remains unclear how typical or atypical antipsychotics affect the BDNF-mediated intracellular Ca^2+^ mobilization in microglia.

There is increasing evidence suggesting that pathophysiology of schizophrenia is related to the inflammatory responses mediated by microglia (Müller and Schwarz, 2007; Kato et al., 2011; Monji et al., 2013; Myint and Kim, 2014). In addition, we have reported that pretreatment with antipsychotics significantly inhibits the release of proinflammatory cytokines and/or NO from activated microglial cells, possibly through the suppression of [Ca^2+^]i elevation in microglial cells (Kato et al., 2008, 2013; Mizoguchi et al., 2011). For electrically non-excitable cells such as microglia, the primary source of intracellular Ca^2+^ is the ER. suggesting the importance of the ER as a therapeutic target of antipsychotics for the treatment of schizophrenia.

## Conclusion

Microglia can release many factors including proinflammatory cytokines, NO and BDNF when they are activated in response to immunological stimuli. There is increasing evidence suggesting that pathophysiology of schizophrenia is related to the inflammatory responses mediated by microglia. In addition, we have previously reported that pretreatment with antipsychotics significantly inhibits the release of proinflammatory cytokines and/or NO from activated microglial cells, possibly through the suppression of the elevation of [Ca^2+^]i, suggesting the importance of microglial intracellular Ca^2+^ signaling as a target of antipsychotics for the treatment of schizophrenia. Although the electrical activity of neurons mainly depends on voltage-gated calcium channels and NMDARs, the generation of intracellular Ca^2+^ signaling in non-excitable cells such as microglia is mainly regulated by the ER. These suggest the importance of the ER as a therapeutic target of antipsychotics for the treatment of schizophrenia.

## Conflict of interest statement

The authors declare that the research was conducted in the absence of any commercial or financial relationships that could be construed as a potential conflict of interest.

## References

[B1] Aguzzi A. Barres B. A. Bennett M. L. (2013). Microglia: scapegoat, saboteur, or something else? Science 339, 156–161. 10.1126/science.1227901 23307732 PMC4431634

[B2] Autry A. E. Monteggia L. M. (2012). Brain-derived neurotrophic factor and neuropsychiatric disorders. Pharmacol. Rev. 64, 238–258. 10.1124/pr.111.005108 22407616 PMC3310485

[B3] Bai J. He F. Novikova S. I. Undie A. S. Dracheva S. Haroutunian V.. (2004). Abnormalities in the dopamine system in schizophrenia may lie in altered levels of dopamine receptor-interacting proteins. Biol. Psychiatry 56, 427–440. 10.1016/j.biopsych.2004.06.022 15364041

[B4] Berridge M. J. (2013). Dysregulation of neural calcium signaling in Alzheimer disease, bipolar disorder and schizophrenia. Prion 7, 2–13. 10.4161/pri.21767 22895098 PMC3609045

[B5] Berridge M. J. Bootman M. D. Roderick H. L. (2003). Calcium signalling: dynamics, homeostasis and remodelling. Nat. Rev. Mol. Cell Biol. 4, 517–529. 10.1038/nrm1155 12838335

[B6] Choi K. H. Rhim H. (2010). Inhibition of recombinant Ca(v)3.1 (alpha(1G)) T-type calcium channels by the antipsychotic drug clozapine. Eur. J. Pharmacol. 626, 123–130. 10.1016/j.ejphar.2009.09.035 19782679

[B7] Cunningham C. (2013). Microglia and neurodegeneration: the role of systemic inflammation. Glia 61, 71–90. 10.1002/glia.22350 22674585

[B8] Färber K. Kettenmann H. (2006). Functional role of calcium signals for microglial function. Glia 54, 656–665. 10.1002/glia.20412 17006894

[B9] Green M. J. Matheson S. L. Shepherd A. Weickert C. S. Carr V. J. (2011). Brain-derived neurotrophic factor levels in schizophrenia: a systematic review with meta-analysis. Mol. Psychiatry 16, 960–972. 10.1038/mp.2010.88 20733577

[B10] Hou Y. Wu C. F. Yang J. Y. He X. Bi X. L. Yu L.. (2006). Effects of clozapine, olanzapine and haloperidol on nitric oxide production by lipopolysaccharide-activated N9 cells. Prog. Neuropsychopharmacol. Biol. Psychiatry 30, 1523–1528. 10.1016/j.pnpbp.2006.05.006 16806626

[B11] Jimerson D. C. Post R. M. Carman J. S. van Kammen D. P. Wood J. H. Goodwin F. K.. (1979). CSF calcium: clinical correlates in affective illness and schizophrenia. Biol. Psychiatry 14, 37–51. 420907

[B12] Kato T. Mizoguchi Y. Monji A. Horikawa H. Suzuki S. O. Seki Y.. (2008). Inhibitory effects of aripiprazole on interferon-gamma-induced microglial activation via intracellular Ca^2+^ regulation in vitro. J. Neurochem. 106, 815–825. 10.1111/j.1471-4159.2008.05435.x 18429930

[B13] Kato T. Monji A. Hashioka S. Kanba S. (2007). Risperidone significantly inhibits interferon-gamma-induced microglial activation in vitro. Schizophr. Res. 92, 108–115. 10.1016/j.schres.2007.01.019 17363222

[B14] Kato T. A. Monji A. Yasukawa K. Mizoguchi Y. Horikawa H. Seki Y.. (2011). Aripiprazole inhibits superoxide generation from phorbol-myristate-acetate (PMA)-stimulated microglia in vitro: implication for antioxidative psychotropic actions via microglia. Schizophr. Res. 129, 172–182. 10.1016/j.schres.2011.03.019 21497059

[B15] Kato T. A. Yamauchi Y. Horikawa H. Monji A. Mizoguchi Y. Seki Y.. (2013). Neurotransmitters, psychotropic drugs and microglia: clinical implications for psychiatry. Curr. Med. Chem. 20, 331–344. 10.2174/0929867311320030003 23157624

[B16] Kettenmann H. Hanisch U. K. Noda M. Verkhratsky A. (2011). Physiology of microglia. Physiol. Rev. 91, 461–553. 10.1152/physrev.00011.2010 21527731

[B17] Kurosawa S. Hashimoto E. Ukai W. Toki S. Saito S. Saito T. (2007). Olanzapine potentiates neuronal survival and neural stem cell differentiation: regulation of endoplasmic reticulum stress response proteins. J. Neural Transm. 114, 1121–1128. 10.1007/s00702-007-0747-z 17557129

[B18] Lewis D. A. Hashimoto T. Volk D. W. (2005). Cortical inhibitory neurons and schizophrenia. Nat. Rev. Neurosci. 6, 312–324. 10.1038/nrn1648 15803162

[B19] Lidow M. S. (2003). Calcium signaling dysfunction in schizophrenia: a unifying approach. Brain Res. Brain Res. Rev. 43, 70–84. 10.1016/s0165-0173(03)00203-0 14499463

[B20] Luo X. J. Li M. Huang L. Steinberg S. Mattheisen M. Liang G.. (2014). Convergent lines of evidence support CAMKK2 as a schizophrenia susceptibility gene. Mol. Psychiatry 19, 774–783. 10.1038/mp.2013.103 23958956

[B21] Miller B. J. Buckley P. Seabolt W. Mellor A. Kirkpatrick B. (2011). Meta-analysis of cytokine alterations in schizophrenia: clinical status and antipsychotic effects. Biol. Psychiatry 70, 663–671. 10.1016/j.biopsych.2011.04.013 21641581 PMC4071300

[B22] Mizoguchi Y. Kato T. A. Seki Y. Ohgidani M. Sagata N. Horikawa H.. (2014). BDNF induces sustained intracellular Ca^2+^ elevation through the upregulation of surface TRPC3 channels in rodent microglia. J. Biol. Chem. 289, 18549–18555. 10.1074/jbc.M114.555334 24811179 PMC4140290

[B23] Mizoguchi Y. Monji A. Kato T. A. Horikawa H. Seki Y. Kasai M.. (2011). Possible role of BDNF-induced microglial intracellular Ca(2+) elevation in the pathophysiology of neuropsychiatric disorders. Mini. Rev. Med. Chem. 11, 575–581. 10.2174/138955711795906932 21699488

[B24] Mizoguchi Y. Monji A. Kato T. Seki Y. Gotoh L. Horikawa H.. (2009). Brain-derived neurotrophic factor induces sustained elevation of intracellular Ca^2+^ in rodent microglia. J. Immunol. 183, 7778–7786. 10.4049/jimmunol.0901326 19923466

[B25] Möller T. (2002). Calcium signaling in microglial cells. Glia 40, 184–194. 10.1002/glia.10152 12379906

[B26] Monji A. Kato T. A. Mizoguchi Y. Horikawa H. Seki Y. Kasai M.. (2013). Neuroinflammation in schizophrenia especially focused on the role of microglia. Prog. Neuropsychopharmacol. Biol. Psychiatry 42, 115–121. 10.1016/j.pnpbp.2011.12.002 22192886

[B27] Müller N. Schwarz M. J. (2007). The immune-mediated alteration of serotonin and glutamate: towards an integrated view of depression. Mol. Psychiatry 12, 988–1000. 10.1038/sj.mp.4002006 17457312

[B28] Myint A. M. Kim Y. K. (2014). Network beyond IDO in psychiatric disorders: revisiting neurodegeneration hypothesis. Prog. Neuropsychopharmacol. Biol. Psychiatry 48, 304–313. 10.1016/j.pnpbp.2013.08.008 24184687

[B29] Nagano T. Kimura S. H. Takai E. Matsuda T. Takemura M. (2006). Lipopolysaccharide sensitizes microglia toward Ca^2+^-induced cell death: mode of cell death shifts from apoptosis to necrosis. Glia 53, 67–73. 10.1002/glia.20260 16158419

[B30] Nilius B. Owsianik G. Voets T. Peters J. A. (2007). Transient receptor potential cation channels in disease. Physiol. Rev. 87, 165–217. 10.1152/physrev.00021.2006 17237345

[B31] Orrenius S. Zhivotovsky B. Nicotera P. (2003). Regulation of cell death: the calcium-apoptosis link. Nat. Rev. Mol. Cell Biol. 4, 552–565. 10.1038/nrm1150 12838338

[B32] Parekh A. B. Putney J. W. Jr. (2005). Store-operated calcium channels. Physiol. Rev. 85, 757–810. 10.1152/physrev.00057.2003 15788710

[B33] Parkhurst C. N. Yang G. Ninan I. Savas J. N. Yates J. R. 3rd Lafaille J. J.. (2013). Microglia promote learning-dependent synapse formation through brain-derived neurotrophic factor. Cell 155, 1596–1609. 10.1016/j.cell.2013.11.030 24360280 PMC4033691

[B34] Reuss B. Unsicker K. (2001). Atypical neuroleptic drugs downregulate dopamine sensitivity in rat cortical and striatal astrocytes. Mol. Cell. Neurosci. 18, 197–209. 10.1006/mcne.2001.1017 11520180

[B35] Ripke S. O’Dushlaine C. Chambert K. Moran J. L. Kähler A. K. Akterin S.. (2013). Genome-wide association analysis identifies 13 new risk loci for schizophrenia. Nat. Genet. 45, 1150–1159. 10.1038/ng.2742 23974872 PMC3827979

[B36] Santi C. M. Cayabyab F. S. Sutton K. G. McRory J. E. Mezeyova J. Hamming K. S.. (2002). Differential inhibition of T-type calcium channels by neuroleptics. J. Neurosci. 22, 396–403. 11784784 10.1523/JNEUROSCI.22-02-00396.2002 PMC6758663

[B37] Schwartz M. Kipnis J. Rivest S. Prat A. (2013). How do immune cells support and shape the brain in health, disease and aging? J. Neurosci. 33, 17587–17596. 10.1523/JNEUROSCI.3241-13.2013 24198349 PMC3818540

[B38] Simms B. A. Zamponi G. W. (2014). Neuronal voltage-gated calcium channels: structure, function and dysfunction. Neuron 82, 24–45. 10.1016/j.neuron.2014.03.016 24698266

[B39] Smith A. M. Dragunow M. (2014). The human side of microglia. Trends Neurosci. 37, 125–135. 10.1016/j.tins.2013.12.001 24388427

[B40] Smoller J. W. Ripke S. Lee P. H. Neale B. Nurnberger J. I. Santangelo S.. (2013). Identification of risk loci with shared effects on five major psychiatric disorders: a genome-wide analysis. Lancet 381, 1371–1379. 10.1016/S0140-6736(12)62129-1 23453885 PMC3714010

[B41] Solís-Chagoyán H. Calixto E. Figueroa A. Montaño L. M. Berlanga C. Rodríguez-Verdugo M. S.. (2013). Microtubule organization and L-type voltage-activated calcium current in olfactory neuronal cells obtained from patients with schizophrenia and bipolar disorder. Schizophr. Res. 143, 384–389. 10.1016/j.schres.2012.11.035 23290267

[B42] Takano A. Arakawa R. Ito H. Tateno A. Takahashi H. Matsumoto R.. (2010). Peripheral benzodiazepine receptors in patients with chronic schizophrenia: a PET study with [11C]DAA1106. Int. J. Neuropsychopharmacol. 13, 943–950. 10.1017/S1461145710000313 20350336

[B43] Tan Q. R. Wang X. Z. Wang C. Y. Liu X. J. Chen Y. C. Wang H. H.. (2007). Differential effects of classical and atypical antipsychotic drugs on rotenone-induced neurotoxicity in PC12 cells. Eur. Neuropsychopharmacol. 17, 768–773. 10.1016/j.euroneuro.2007.03.003 17442543

[B44] Thastrup O. Cullen P. J. Drøbak B. K. Hanley M. R. Dawson A. P. (1990). Thapsigargin, a tumor promoter, discharges intracellular Ca^2+^ stores by specific inhibition of the endoplasmic reticulum Ca2(+)-ATPase. Proc. Natl. Acad. Sci. U S A 87, 2466–2470. 10.1073/pnas.87.7.2466 2138778 PMC53710

[B45] van Berckel B. N. Bossong M. G. Boellaard R. Kloet R. Schuitemaker A. Caspers E.. (2008). Microglia activation in recent-onset schizophrenia: a quantitative (R)-[11C]PK11195 positron emission tomography study. Biol. Psychiatry 64, 820–822. 10.1016/j.biopsych.2008.04.025 18534557

[B46] Wake H. Moorhouse A. J. Miyamoto A. Nabekura J. (2013). Microglia: actively surveying and shaping neuronal circuit structure and function. Trends Neurosci. 36, 209–217. 10.1016/j.tins.2012.11.007 23260014

[B47] Zhang K. Kaufman R. J. (2008). From endoplasmic-reticulum stress to the inflammatory response. Nature 454, 455–462. 10.1038/nature07203 18650916 PMC2727659

